# Synergistic Antibiofilm Efficacy of Thymol and Piperine in Combination with Three Aminoglycoside Antibiotics against *Klebsiella pneumoniae* Biofilms

**DOI:** 10.1155/2021/7029944

**Published:** 2021-11-08

**Authors:** Borel Bisso Ndezo, Christian Ramsès Tokam Kuaté, Jean Paul Dzoyem

**Affiliations:** Laboratory of Microbiology and Antimicrobial Substances, Department of Biochemistry, Faculty of Science, University of Dschang, Dschang, Cameroon

## Abstract

**Background:**

Thymol and piperine are two naturally occurring bioactive compounds with several pharmacological activities. In this study, their antibiofilm potential either alone or in combination with three aminoglycoside antibiotics was evaluated against a biofilm of *Klebsiella pneumoniae*.

**Methods:**

Determination of antimicrobial susceptibility was performed using the broth microdilution method. Biofilm formation was evaluated by the microtiter plate method. Antibiofilm activity was determined using 3-(4, 5-dimethyl-2-thiazolyl)-2, 5-diphenyl-2H-tetrazolium-bromide (MTT) assay. The combination studies were performed by the checkerboard microdilution method.

**Results:**

The minimum biofilm inhibitory concentration (MBIC) of streptomycin was reduced by 16- to 64-fold when used in combination with thymol, while the MBIC of kanamycin was reduced by 4-fold when combined with piperine. The minimum biofilm eradication concentration (MBEC) values of streptomycin, amikacin, and kanamycin were, respectively, 16- to 128-fold, 4- to 128-fold, and 8- to 256-fold higher than the planktonic minimum inhibitory concentration (MIC). Thymol combined with streptomycin or kanamycin showed synergic effects against the preformed biofilm with 16- to 64-fold reduction in the minimum biofilm eradication concentration values of each antibiotic in combination. Piperine acted also synergically with kanamycin with an 8- to 16-fold reduction in the minimum biofilm eradication concentration values of kanamycin in combination.

**Conclusion:**

The association of thymol with antibiotics showed a strong synergistic effect both in the inhibition of biofilm formation and the destruction of the preformed biofilm of *K. pneumoniae*. This study suggests that a combination of thymol with streptomycin, amikacin, or kanamycin could be a promising alternative therapy to overcome the problem of *K. pneumoniae* biofilm-associated infections.

## 1. Introduction

The emergence and spread of resistant bacteria have become a serious health concern contributing to a high rate of mortality and morbidity. *Klebsiella pneumoniae is* one of the most common Gram-negative bacteria associated with nosocomial infections, respiratory infections, urinary tract infections, liver abscesses, and bacteremia [1]. Its ability to adhere and grow as a biofilm on mucous membranes is crucial for the progression of infection. Studies reported that growth as a biofilm promotes multidrug resistance to antibiotics and high resistance to the immune system [2].

Biofilms are aggregates of microorganisms in which cells are attached to a biotic or abiotic surface and surrounded by a protective and adhesive extracellular matrix of exopolysaccharides, extracellular DNA, and proteins [3]. Biofilm formation is a complex cooperative group process, which occurs in step-by-step processes and involves chemical communication within and between cells. Biofilm formation proceeds in four stages: adhesion, microcolony formation, maturation, and dispersion [4].

It is estimated that 80% of chronic and recurrent bacterial infections are due to the ability of bacteria to form biofilms. In addition, biofilm bacteria are 10- to 1000-fold resistant to conventional antibiotics normally used to treat planktonic cells [5]. The infections associated with biofilms are difficult to treat because of slow penetration or sequestration of antimicrobial agents into the biofilm through the extracellular matrix, the presence of multidrug-resistant persister cells, and the low metabolic state at the base of the biofilm [6]. Therefore, there is an urgent need for the identification of new approaches to a therapeutic formulation that targets the biofilm mode of growth to prevent or treat biofilm-related infections. Drug combinations have been used as an alternative to effectively combat biofilm. Such an approach includes antibiotic-antibiotic combinations and the association of an antibiotic with a nonantibiotic adjuvant molecule to directly target resistance mechanisms. It is necessary to develop new antimicrobial drugs which are effective against bacterial biofilms through the combination of various active agents.

Plant-derived natural products as adjuvant molecules have been studied for their potential for biofilm reduction [7]. A large body of evidence highlighted the beneficial effect of using bioactive natural products in conjunction with conventional antibiotics, leading to enhanced activity against bacterial biofilms. For instance, the antibiofilm efficacy of gentamicin and trimethoprim was significantly increased in the presence of the bioactive compound gallotannin [8]. So, natural compounds can be alternatively used in combination with antibiotics to enhance the activity against biofilm-related infection.

Piperine, a naturally occurring alkaloid is the major bioactive component responsible for the pungency of commonly consumed spices black pepper or white pepper (*Piper nigrum*) and long pepper (*Piper longum*). Thymol is a natural volatile monoterpenoid phenol that is the main active ingredient of oil extracted from species *Thymus vulgaris* L., *Ocimum gratissimum* L., *Origanum* L., and *Carum copticum* L. Piperine and thymol are versatile molecules with a wide variety of pharmacological activities including antibacterial and antibiofilm activities [9, 10].

However, the sole use of these natural agents alone has limited antimicrobial activity. Therefore, the present study aimed to investigate the antibiofilm activity of thymol and piperine in combination with aminoglycosides against the *K. pneumoniae* biofilm.

## 2. Materials and Methods

### 2.1. Chemical and Natural Products

Antibiotics used (streptomycin, amikacin, and kanamycin) as well as natural products thymol (purity ≥98.5%) and piperine (purity ≥97%) were purchased from Sigma-Aldrich. Similarly, dimethyl sulfoxide (DMSO), p-iodonitrotetrazolium chloride (INT), and 3-(4,5-dimethythiazole-2-yl)-2,5-diphenyltetrazolium bromide (MTT) were purchased from Sigma-Aldrich. The Muller Hinton Agar (MHA) and Muller Hinton Broth (MHB) media were purchased from Dominique Dutscher SAS, France.

### 2.2. Microorganisms

Four clinical isolates of *K. pneumoniae* isolated from urine samples, namely, *Kp02*, *Kp03*, *Kp04,* and *Kp05*, were obtained from the Laboratory of Microbiology of the “*Université des Montagnes,*” West Cameroon (courtesy of Dr. Fotsing Pierre). Another clinical isolate *Kp55* was a courtesy of Prof. Kuété Victor from the Laboratory of Microbiology and Natural Substances of the University of Dschang, Cameroon.

### 2.3. Minimum Inhibitory Concentration (MIC) and Minimum Bactericidal Concentration (MBC) Determination of Thymol, Piperine, and Antibiotics

The susceptibility of *K. pneumoniae* isolates towards thymol, piperine, and antibiotics was assessed by determining MICs and MBCs. The broth microdilution method was used as previously described [11, 12]. Briefly, 100 *μ*L of bacterial inoculum (1.5 × 10^6^ CFU/mL) was incubated with serial two-fold dilutions of 100 *μ*L of antibiotic or natural product for 24 h at 37°C. Concentrations ranged from 256 to 0.125 *μ*g/mL for antibiotics and 1024 to 0.5 *μ*g/mL for natural products. Untreated wells served as the positive control. After incubation, the presence of bacterial growth was detected by adding to each well 40 *μ*L of *p*-iodonitrotetrazolium chloride (INT) (0.2 mg/mL), and plates were incubated again at 37°C for 30 min. Viable bacteria reduced the yellow dye to pink. MIC was recorded as the lowest concentration that prevented the color change of the medium. MBC was determined by adding 50 *μ*L aliquots from the wells that did not show growth after incubation for the MIC test to 150 *μ*L of MHB. After incubation at 37°C for 48 h, the MBC was recorded as the lowest concentration of the antibiotic or natural product, which did not observe a color change after the addition of INT solution as described above. The experiment was performed in triplicate and repeated three times.

### 2.4. Kinetics of Biofilm Formation and Its Metabolic Activity

Biofilm formation ability of the isolates was evaluated by the microtiter plate method as described by Kirmusaoğlu and Kaşıkçı with slight modifications [13]. Briefly, 200 *μ*L of bacterial suspension (7.5 × 10^5^ CFU/mL) in MHB supplemented with 2% glucose was added into 96-well flat-bottomed sterile polystyrene microplate and incubated at 37°C for 6 h, 12 h, 24 h, 48 h, and 72 h. At the end of each time point, the media in wells of the microplate were discharged, and the plate was washed three times with phosphate-buffered saline (PBS; pH 7.2) to remove nonadherent bacterial cells. The wells containing MHB without bacteria served as the negative control. The cells that attached to the well surface were considered as a true biofilm. The biofilm formation was measured using MTT reduction assay. In brief, the volume of 200 *μ*L of MTT (0.5 mg/mL) prepared in PBS was added to each well, and the plate was incubated at 37°C for 4 h. After incubation, the solution from wells was removed and 150 *μ*L of DMSO was added to the plate to dissolve the formazan crystal formed by viable bacteria. The absorbance of plates was measured at 570 nm using a microplate reader (Spectramax 190, Molecular Devices).

### 2.5. Biofilm Inhibition Assay

The capacity of thymol, piperine, and antibiotics to prevent the formation of biofilm by *K. pneumoniae* was assessed according to the work of Teanpaisan et al. [14]. The plates were filled with 100 *μ*L of inoculum (1.5 × 10^6^ CFU/mL) and 100 *μ*L of concentration of natural products or antibiotics. Concentrations ranged from 0.25 to 512 *μ*g/mL for antibiotics and from 1 to 2048 *μ*g/mL for thymol and piperine. The plates were incubated for 24 h at 37°C. After incubation, the plates were treated for the biofilm formation assay as described above. Untreated wells were used as the positive control, and the wells containing MHB broth without bacteria were used as blank. The percentage cell viability inhibition was calculated using the following formula:

(1) $$\begin{array}{c} \%\text{ cell viability inhibition}=\frac{\left(OD_{570}\text{ control}-OD_{570}\text{blank }\right)-\left(OD_{570}\text{test}-OD_{570}\text{blank}\right)}{\left(OD_{570}\text{control}-OD_{570}\text{blank}\right)}\;\times 100. \end{array}$$

The minimal biofilm inhibitory concentration (MBIC) was recorded as the lowest concentration of natural products or antibiotics reducing the biofilm metabolic activity by 100%.

### 2.6. Biofilm Eradication Assay

To assess the capacity of the thymol, piperine, and antibiotics to eradicate biofilms, the microtiter plate method was used [13]. After biofilm formation for 48 h, the medium was discarded, and the wells were carefully washed with PBS to remove nonadherent bacteria cells. Then, the plates were filled with 100 *μ*L of MHB supplemented with 2% glucose and 100 *μ*L of natural products or antibiotics, at concentrations ranging from 1 to 2048 *μ*g/mL and 0.25 to 512 *μ*g/mL, respectively. The plates were incubated at 37°C for 24 h. After incubation, the plates were treated for the biofilm formation assay as described above. The minimal biofilm eradication concentration (MBEC) was defined as the lowest concentration of natural products or antibiotics reducing the metabolic activity in the preformed biofilm by 100%. The experiment was performed in triplicate and repeated three times.

### 2.7. Thymol and Piperine in Combination with Antibiotics against the Biofilm Formation

The interactions of thymol/piperine and antibiotics to prevent the formation of biofilm of *K*. *pneumoniae* were assessed using the broth microdilution checkerboard method according to previously described protocols with minor changes [15]. In brief, in 96-well flat-bottomed polystyrene plates containing 50 *μ*L of MHB supplemented with 2% glucose, the antibiotic (50 *μ*L) was serially two-fold diluted horizontally while the natural product (50 *μ*L) was serially two-fold diluted vertically. Then, 100 *μ*L of bacterial inoculum (1.5 × 10^6^ CFU/mL) was added to each well, and plates were incubated at 37°C for 24 h. The final concentrations ranged from 0.625 to 64 *μ*g/mL for antibiotics and from 16 to 1024 *μ*g/mL for thymol and piperine. After incubation, the broth in wells was gently removed and the plates were washed three times with PBS. Untreated wells were used as the positive control, and wells containing MHB without bacteria were used as blank. MTT reduction assay as mentioned above was used to assess the metabolic activity in the biofilm, and minimum biofilm inhibitory concentration (MBIC) was determined. The effect of combinations was determined by calculating the fractional inhibitory concentration index (FICI) using the following equation: FICI = (MBIC of the antibiotic in the combination/MBIC of the antibiotic alone) + (MBIC of the natural product in the combination/MBIC of the natural product alone). The type of interaction between natural products and antibiotics was defined based on the FICI value as follows: synergy when FICI ≤ 0.5, additivity when 0.5 < FICI ≤ 1, indifference when 1 < FICI ≤ 4, and antagonism when FICI > 4 [15].

### 2.8. Thymol and Piperine in Combination with Antibiotics against the Preformed Biofilm

To evaluate the effect of the combination of thymol and piperine with antibiotics to eradicate the biofilm of K. *pneumoniae*, the checkerboard method was used as described above with minor modifications [15]. In effect, after biofilm formation for 48 h, the nonadherent cells were gently removed and the plate was washed with PBS three times. Then, 100 *μ*L of MHB supplemented with 2% glucose and 50 *μ*L of each substance as described above were added to the adherent cells into the wells at final concentrations of 0.625–64 *μ*g/mL for antibiotic and 16–1024 *μ*g/mL for the natural product. The wells containing the medium and bacteria were used as the positive control, while wells containing MHB were used as blank. After incubation for 24 h at 37°C, the MTT reduction assay as mentioned above was performed to evaluate the metabolic activity of the biofilms and the minimum biofilm eradication concentration (MBEC) was determined. The FICI was calculated and interpreted as described above.

### 2.9. Statistical Analysis

Data were analyzed using GraphPad Prism 8.0. Results were expressed as means ± standard deviations (SDs) of three independent experiments. The significance of differences between interval times of the biofilm quantification was determined using Fisher's least significant difference (LSD) at 5% significance level.

## 3. Results

### 3.1. Susceptibility of *K. pneumoniae* Planktonic Cells to Thymol, Piperine, and Antibiotics

The susceptibility of *K*. *pneumoniae* planktonic cells against thymol, piperine amikacin, kanamycin, and streptomycin is shown in Table 1. MICs values varied from 64 to 256 *μ*g/mL, 512 to 1024 *μ*g/mL, and 0.25 to 16 *μ*g/mL for thymol, piperine, and antibiotics, respectively. MBC values were, respectively, in the range of 256–512 *μ*g/mL, 1024->1024 *μ*g/mL, and 2–64 *μ*g/mL for thymol, piperine, and antibiotics.

### 3.2. Kinetics of the Biofilm Formation by *K. pneumoniae* Isolates

**Figure 1:**
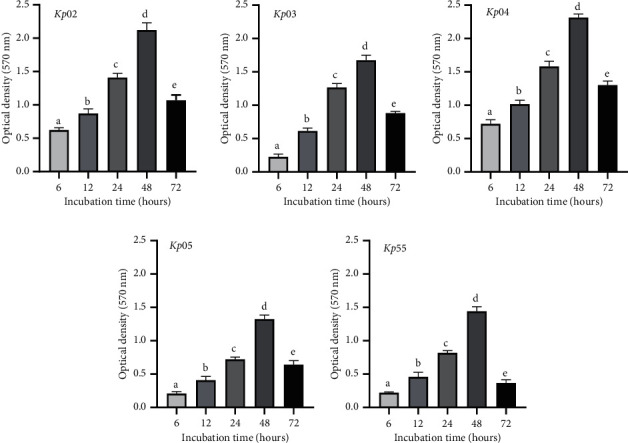
Kinetics of the biofilm-producing capacity of *K. pneumoniae* clinical isolates. Clinical isolates of *K*. *pneumoniae* were incubated for 72 h, and the biofilm was analyzed at 6, 12, 24, 48, and 72 h to determine the best time for maximum biofilm formation. The bar graphs represent the mean (*n* = 3) ± standard deviation (error bars) of the values corresponding to each time of incubation for biofilm formation. Different letters (a, b, c d, and e) above bars indicate a significant difference between the values (*p* < 0.05 according to Fisher's least significant difference).

The biofilm formation kinetics was performed up to 72 h, and the mean optical density values at 570 nm were plotted against time (Figure 1). Data show that, for all *K*. *pneumoniae* isolates evaluated, the highest quantity of biofilm was obtained after 48 h incubation with optical densities (ODs) values ranging from 1.32 to 2.31. *Kp02* (OD = 2.13) and *Kp04* (OD = 2.31) were the best biofilm-forming isolates. However, the biofilm formation decreased after 72 h incubation with the OD values decreasing from 2.31 to 1.3 and from 2.12 to 1.06 for *Kp02* and *Kp04*, respectively.

### 3.3. Combination Interaction of Thymol and Piperine with Aminoglycoside Antibiotics against Biofilm Formation

The antibiofilm efficacy of thymol, piperine, and three aminoglycosides (amikacin, kanamycin, and streptomycin) alone against *K*. *pneumoniae* was performed in terms of biofilm formation and dispersal, and MBIC and MBEC values were determined, respectively. In a combination study, the FIC index was used to appreciate the interaction between natural products and antibiotics. The result of the potential of thymol, piperine, and antibiotics to prevent the formation of biofilm in *K*. *pneumoniae* isolates is presented in Table 2. When tested alone, MBIC values of thymol ranged from 256 to 1024 *μ*g/mL while piperine was 1024 *μ*g/mL. Antibiotics showed MBIC values ranged from 4–8 *μ*g/mL, 2–16 *μ*g/mL, and 1–8 *μ*g/mL for streptomycin, amikacin, and kanamycin, respectively. The effect of the association of natural products with antibiotics against biofilm formation (Table 2) revealed that synergism was observed in all of *K. pneumoniae* isolates when thymol was combined with streptomycin, with FICI values ranging from 0.13 to 0.27 and 16- to 64-fold reduction in the MBIC of streptomycin. Synergistic interaction was found in the combination of thymol with amikacin against *Kp02* (FICI = 0.25) and *Kp04* (FICI = 0.5) isolates with 8-fold and a 4-fold reduction in the MBIC value of amikacin, respectively. In the same combination, additive interaction was observed against *Kp55* (FICI = 0.56) with a 16-fold reduction in the MBIC of amikacin. Thymol acted also synergically with kanamycin against *Kp55* (FICI = 0.28), *Kp02* (FICI = 0.31), and *Kp04* (FICI = 0.16) isolates with 4-fold, 16-fold, and 32-fold reduction, respectively, in the MBIC of kanamycin. A combination of piperine with amikacin showed a synergic effect (FICI = 0.5) against *Kp55* isolate with a 4-fold reduction in the MBIC of amikacin.

MBIC: minimal biofilm inhibitory concentration, ATB: antibiotic, Thy: thymol, Pip: piperine, (ATB/Thy)^*∗*^: concentration of antibiotic in combination with thymol, (Thy)^*∗*^: concentration of thymol in combination with antibiotic, (ATB/Pip)^*∗*^: concentration of antibiotic in combination with piperine, (Pip)^*∗*^: concentration of piperine in combination with antibiotic, (ATB/Thy)^*α*^ = concentration of antibiotic alone/(ATB/Thy)^*∗*^, (ATB/Pip)^*α*^ = concentration of antibiotic alone/(ATB/Pip)^*∗*^, (ATB/Thy)^ο^ =  (ATB/Thy)^*∗*^/concentration of antibiotic alone + (Thy)^*∗*^/concentration of thymol alone, (ATB/Pip)^ο^ =  (ATB/Pip)^*∗*^/concentration of antibiotic alone + (Pip)^*∗*^/concentration of piperine alone, FICI: fractional inhibitory concentration index, S: synergy, Ad: additivity, I: indifference.

### 3.4. Combination Interaction of Thymol and Piperine with Aminoglycoside Antibiotics against Preformed Biofilm

Table 3 shows the capacity of thymol, piperine, and antibiotics to disperse preformed biofilm in *K. pneumoniae* isolates. The tested compounds eradicated the preformed biofilm with MBEC values between 32–128 *μ*g/mL for antibiotics, 512–1024 *μ*g/mL for thymol, and 1024 *μ*g/mL for piperine. In combination, FICI values obtained in the association of thymol with streptomycin ranged from 0.08 to 0.28, indicating the synergic effect (FICI ≤ 0.5). In the same combination, thymol in a concentration of 64–128 *μ*g/mL significantly decreased (16- to 64-fold) the MBEC of streptomycin from 32–128 *μ*g/mL to 0.5–4 *μ*g/mL.

Thymol combined with amikacin showed also a synergic effect against isolates *Kp03* and *Kp05*, reducing 4-fold and 8-fold, respectively, the MBEC of amikacin. With *Kp04* and *Kp05* isolates, the combination of thymol with kanamycin demonstrated the synergy (FICI = 0.27 and 0.26, respectively) reducing 64-fold (from 64 *μ*g/mL to 1 *μ*g/mL) and 16-fold (from 128 *μ*g/mL to 8 *μ*g/mL), respectively, the MBEC of kanamycin. When piperine was used in combination with streptomycin, it showed a synergic effect against *Kp04* isolate and reduced 8-fold (from 64 *μ*g/mL to 8 *μ*g/mL) the MBEC of streptomycin.

Piperine in combination with kanamycin showed synergic effects (FICI = 0.09–0.38) against the mature biofilm of *Kp*55, *Kp04,* and *Kp05* isolates with a reduction in kanamycin MBEC (8-, 16-, and 8-fold, respectively).

## 4. Discussion

In recent years, the emergence of drug-resistant *K. pneumoniae* isolates has increased continuously. Biofilms are generally more resistant to antimicrobial chemotherapies than free-living bacteria. High resistance of biofilm infections to antibiotics and physical treatments is supported by factors such as persistent cells, adaptive responses, limited antibiotic penetration, and genetic factors [16]. Innovative antibiofilm strategies such as the prevention of biofilm formation, dispersion of preformed biofilms, and antibiotic combinations against biofilm-associated infections need to be explored. In this study, we investigated the antibiofilm effect of two naturally occurring compounds thymol and piperine in combination with three aminoglycoside antibiotics (amikacin, kanamycin, and streptomycin) against *K. pneumoniae* clinical isolates.

In a preliminary study, the antibacterial activity of the compounds against *K. pneumoniae* planktonic cells was performed. Both thymol and piperine had an inhibitory effect against planktonic cells of *K. pneumoniae*, with thymol showing lower MICs compared to that of piperine. According to a recent classification, the antibacterial activity of natural product is considered as very good when MIC ≤ 15 *μ*g/mL, good when 15 < MIC ≤ 25 *μ*g/mL, moderate 25 < MIC ≤ 100 *μ*g/mL, and low when MIC > 100 *μ*g/mL [17]. Therefore, thymol showed moderate to low antibacterial while piperine showed low activity against *K. pneumoniae* isolates. The MIC values ranged from 64 to 256 and 512 to 1024 *μ*g/mL for thymol and piperine, respectively. MIC values of thymol and piperine obtained from the present study were comparatively lower than the previously reported ones. El Atki et al. reported the inhibitory effect of thymol on the growth of *K. pneumoniae* at 703 *μ*g/mL [18], while Toma observed an MIC value of 25 mg/mL in the case of piperine against *K. pneumoniae* [19].

Piperine is an alkaloid, and little is known about its antibacterial activity against K. *pneumoniae*. However, the antimicrobial activity of piperine is higher against Gram-positive bacteria as compared to that of Gram-negative bacteria [20]. Despite the high levels of aminoglycoside resistance among *Klebsiella pneumoniae* isolates observed in previous studies [21], all of *K. pneumoniae* planktonic cells isolates were susceptible to streptomycin, amikacin, and kanamycin in this study.

In our study, the kinetics of *K. pneumoniae* isolate biofilm formation revealed that the highest amount of biofilm was produced after 48 h of incubation, followed by a decrease at 72 h incubation. This finding could be correlated to the later stages of the biofilm formation (maturation and dispersion phases) which are characterized by the maturation of biofilm; afterward, some cells start to detach from the mature biofilm (biofilm dispersion) and release free-floating microbes for further colonization [22]. Maldonado et al. observed that the metabolic activity into the biofilm of *K. pneumoniae* increased over time until 24 h of the assay [23].

Thymol inhibited the biofilm formation in *K. pneumoniae* at concentrations ranging from 256 to 1024 *μ*g/mL. These results are in agreement with those obtained by Raei et al. who observed that the MBIC values ranged from 200 to 1600 µg/mL for thymol against Gram-negative bacilli [24]. However, limited information exists on the antibiofilm activity of piperine against *K. pneumoniae*. Dwivedi and Singh reported for piperine an MBIC of 0.0407 mg/mL against *Streptococcus mutans* [25]. Medicinal plants such as T. *vulgaris* are an aromatic plant. Its essential oil contains the thymol, which constitutes the major and more active constituent. Ramos et al. reported the antibacterial and antibiofilm activity of *T. vulgaris* against *K. pneumoniae* [26], while Darsini et al. revealed the antibiofilm activity of *P. nigrum* and *P. longum* with the MBIC value ranged from 0.5 to 2 mg/mL against *Streptococcus pyogenes* biofilms [27].

As expected, *K*. *pneumoniae* biofilms were less sensitive to the tested substances compared to their planktonic counterparts. It was observed that concentrations 1- to 8-fold and 1- to 2-fold higher than those obtained against planktonic cells were needed to inhibit biofilm formation in the case of thymol and piperine, respectively. The MBIC of streptomycin, amikacin, and kanamycin was, respectively, from 2- to 8-fold, 1- to 8-fold, and 0.5- to 4-fold higher than their planktonic MICs. Although the mechanisms by which their antibiofilm effects occur are not known, they might interfere with physiological mechanisms of adherent cells such as autoinducer molecules and the extracellular matrix production [28]. Overall, antibiotics (amikacin, streptomycin, and kanamycin) used in the present study inhibited the biofilm formation of *K. pneumoniae* isolates at lower concentrations (1 to 16 *μ*g/mL) than that of thymol and piperine.

However, combination studies showed that thymol and piperine induce a synergistic effect against biofilm formation when used in association with antibiotics. The concentration of streptomycin was reduced by 16- to 64-fold when used in combination with thymol while the MBIC of amikacin was reduced by 4-fold when combined with piperine. These results highlight the large reduction of the quantities of natural compounds and antibiotics used in association compared to the quantities used alone to inhibit the *K. pneumoniae* biofilm formation.

It was noticed that thymol, piperine, and antibiotics were more effective in inhibiting the biofilm formation but become comparatively less effective in dispersing the preformed biofilm. This result might be due to the recalcitrant nature of the mature biofilm [29]. Thymol has been shown to cause only caused very moderate toxicity [30], and several clinical studies of the coadministration of piperine with various drugs have been performed in humans [31]. Therefore, findings from this study indicate that the combination revealed could be considered as potential safe compounds for therapy against biofilm-related *K. pneumoniae* infections.

## 5. Conclusions

This study has shown the synergistic effect of thymol and piperine and combined with streptomycin, kanamycin, and amikacin on biofilm-associated *K. pneumoniae* pathogens. Synergic effects obtained in the combination of thymol or piperine with the three aminoglycoside antibiotics considered indicate that thymol and piperine are promising candidates for the development of novel antibacterial combination therapies against biofilm-associated infections. Further studies are under consideration to carry out these same tests on other bacterial strains of the Gram-positive or -negative type and to investigate the mechanism of action of these combinations as well as the in vivo model of *K. pneumoniae* biofilms.

## Figures and Tables

**Table 1:** Susceptibility of *K. pneumoniae* planktonic cells against thymol, piperine, and antibiotics.

Isolates	Natural products (*μ*g/mL)				Antibiotics (*μ*g/mL)					
	Thymol		Piperine		Amikacin		Kanamycin		Streptomycin	
	MIC	MBC	MIC	MBC	MIC	MBC	MIC	MBC	MIC	MBC
*Kp02*	128	256	1024	1024	1	4	2	8	1	16
*Kp03*	64	512	512	>1024	1	4	0.25	8	0.5	16
*Kp04*	128	256	1024	>1024	16	32	8	16	8	64
*Kp05*	256	512	512	1024	2	8	16	16	2	16
*Kp055*	128	512	1024	>1024	0.5	4	2	2	2	8

MIC: minimum inhibitory concentration; MBC: minimum bactericidal concentration.

**Table 2:** Minimum biofilm inhibitory concentration (MBIC) of thymol, piperine, aminoglycoside, and fractional inhibitory concentration index (FICI) of combination against *K. pneumoniae.*

	MBIC (*μ*g/mL)							MBIC reduction fold of antibiotics		FICI/interpretation	
	Alone			Combined^*∗*^				(ATB/Thy)^*α*^	(ATB/Pip)^*α*^	(ATB/Thy)^ο^	(ATB/Pip)^ο^
	ATB	Thy	Pip	(ATB/Thy)^*∗*^	(Thy)^*∗*^	(ATB/Pip)^*∗*^	Pip^*∗*^				
Streptomycin											
*Kp55*	8	1024	1024	0.125	128	4	128	64	2	0.14/S	0.63/Ad
*Kp02*	4	512	1024	0.25	32	4	256	16	1	0.13/S	1.25/I
*Kp03*	4	512	1024	0.25	64	1	512	16	4	0.19/S	0.75/Ad
*Kp04*	4	512	1024	0.25	32	2	256	16	2	0.13/S	0.75/Ad
*Kp05*	8	256	1024	0.125	64	4	512	64	2	0.27/S	1/Ad
Amikacin											
*Kp55*	4	1024	1024	0.25	64	1	256	16	4	0.13/S	0.50/S
*Kp02*	2	512	1024	0.25	64	1	512	8	2	0.25/S	1/Ad
*Kp03*	4	512	1024	2	256	4	64	2	1	1/Ad	1.06/I
*Kp04*	16	512	1024	4	128	16	16	4	1	0.50/S	1.02/I
*Kp05*	8	256	1024	4	256	8	512	2	1	1.50/I	1.5/I
Kanamycin											
*Kp55*	8	1024	1024	2	32	4	512	4	2	0.28/S	1/Ad
*Kp02*	4	512	1024	0.25	128	4	16	16	1	0.31/S	1.02/I
*Kp03*	1	512	1024	1	32	1	128	1	1	1.06/I	1.13/I
*Kp04*	8	512	1024	0.25	64	2	1024	32	4	0.16/S	1.25/I
*Kp05*	8	256	1024	2	256	4	32	4	2	1.25/I	0.53/Ad

**Table 3:** Minimum biofilm eradication concentration (MBEC) of thymol, piperine, aminoglycoside, and fractional inhibitory concentration index (FICI) of combination against *K. pneumoniae.*

	MBEC (*μ*g/mL)							MBIC reduction fold of antibiotics		FICI/interpretation	
	Alone			Combined^∗^				(ATB/Thy)^*α*^	(ATB/Pip)^*α*^	(ATB/Thy)^ο^	(ATB/Pip)^ο^
	ATB	Thy	Pip	(ATB/Thy)^*∗*^	(Thy)^*∗*^	(ATB/Pip)^*∗*^	Pip^*∗*^				
Streptomycin											
*Kp55*	32	1024	1024	0.5	64	32	256	64	1	0.08/S	1.25/I
*Kp02*	32	1024	1024	0.5	64	16	128	64	2	0.08/S	0.63/Ad
*Kp03*	64	512	1024	4	64	32	512	16	2	0.19/S	1/Ad
*Kp04*	64	512	1024	2	128	8	256	32	8	0.28/S	0.38/S
Kp05	128	512	1024	4	128	64	512	32	2	0.28/S	1/Ad
Amikacin											
*Kp55*	32	1024	1024	32	256	16	1024	1	2	1.25/I	1.50/I
*Kp02*	64	1024	1024	16	1024	64	512	4	1	1.25/I	1.5/I
*Kp03*	128	512	1024	32	128	32	512	4	4	0.50/S	0.75/Ad
*Kp04*	64	512	1024	32	256	64	1024	2	1	1/Ad	2/I
*Kp05*	64	512	1024	8	64	32	1024	8	2	0.25/S	1.5/I
Kanamycin											
*Kp55*	64	1024	1024	64	16	8	128	1	8	1.02/I	0.25/S
*Kp02*	32	1024	1024	16	512	16	256	2	2	1/Ad	0.75/Ad
*Kp03*	64	512	1024	32	128	32	512	2	2	0.75/Ad	1/Ad
*Kp04*	64	512	1024	1	128	4	32	64	16	0.27/S	0.09/S
*Kp05*	64	512	1024	8	64	16	128	16	8	0.25/S	0.38/S

MBEC: minimal biofilm eradication concentration, ATB: antibiotic, Thy: thymol, Pip: piperine, (ATB/Thy)^*∗*^: concentration of antibiotic in combination with thymol, (Thy)^*∗*^: concentration of thymol in combination with antibiotic, (ATB/Pip)^*∗*^: concentration of antibiotic in combination with piperine, (Pip)^*∗*^: concentration of piperine in combination with antibiotic, (ATB/Thy)^*α*^ = concentration of antibiotic alone/(ATB/Thy)^*∗*^, (ATB/Pip)^*α*^ = concentration of antibiotic alone/(ATB/Pip)^*∗*^, (ATB/Thy)^ο^ = (ATB/Thy)^*∗*^/concentration of antibiotic alone + (Thy)^*∗*^/concentration of thymol alone, (ATB/Pip)^ο^ = (ATB/Pip)^*∗*^/concentration of antibiotic alone + (Pip)^*∗*^/concentration of piperine alone, FICI: fractional inhibitory concentration index, S: synergy, Ad: additivity, I: indifference.

## Data Availability

The data used to support the findings of this study are available upon reasonable request from the corresponding author.

## References

[1] Osman E. A., El-Amin N. E., Al-Hassan L. L., Mukhtar M. (2021). Multiclonal spread of *Klebsiella pneumoniae* across hospitals in Khartoum, Sudan. *Journal of Global Antimicrobial Resistance*.

[2] Karimi K., Zarei O., Sedighi P., Taheri M., Doosti-Irani A., Shokoohizadeh L. (2021). Investigation of antibiotic resistance and biofilm formation in clinical isolates of *Klebsiella pneumoniae*. *International Journal of Microbiology*.

[3] de Oliveira Júnior N. G., Franco O. L. (2020). Promising strategies for future treatment of *Klebsiella pneumoniae* biofilms. *Future Microbiology*.

[4] Blackman L. D., Qu Y., Cass P., Locock K. E. S. (2021). Approaches for the inhibition and elimination of microbial biofilms using macromolecular agents. *Chemical Society Reviews*.

[5] Vishwakarma A., Dang F., Ferrell A., Barton H. A., Joy A. (2021). Peptidomimetic polyurethanes inhibit bacterial biofilm formation and disrupt surface established biofilms. *Journal of the American Chemical Society*.

[6] Dostert M., Trimble M. J., Hancock R. E. W. (2021). Antibiofilm peptides: overcoming biofilm-related treatment failure. *RSC Advances*.

[7] Mishra R., Panda A. K., De Mandal S., Shakeel M., Bisht S. S., Khan J. (2020). Natural anti-biofilm agents: strategies to control biofilm-forming pathogens. *Frontiers in Microbiology*.

[8] Bag A., Chattopadhyay R. R. (2017). Synergistic antibiofilm efficacy of a gallotannin 1,2,6-tri-O-galloyl-*β*-D-glucopyranose from *Terminalia chebula* fruit in combination with gentamicin and trimethoprim against multidrug resistant uropathogenic *Escherichia coli* biofilms. *PLoS One*.

[9] Haq I. U., Imran M., Nadeem M., Tufail T., Gondal T. A., Mubarak M. S. (2021). Piperine: a review of its biological effects. *Phytotherapy Research*.

[10] Escobar A., Pérez M., Romanelli G., Blustein G. (2020). Thymol bioactivity: a review focusing on practical applications. *Arabian Journal of Chemistry*.

[11] Dzoyem J. P., Tchuenguem R. T., Kuiate J. R., Teke G. N., Kechia F. A., Kuete V. (2014). In vitro and in vivo antifungal activities of selected cameroonian dietary spices. *BMC Complementary and Alternative Medicine*.

[12] Dzoyem J. P., McGaw L. J., Eloff J. N. (2014). In vitro antibacterial, antioxidant and cytotoxic activity of acetone leaf extracts of nine under-investigated Fabaceae tree species leads to potentially useful extracts in animal health and productivity. *BMC Complementary and Alternative Medicine*.

[13] Kırmusaoğlu S., Kaşıkçı H. (2020). Identification of *ica*-dependent biofilm production by *Staphylococcus aureus* clinical isolates and antibiofilm effects of ascorbic acid against biofilm production. *Journal of Clinical Pathology*.

[14] Teanpaisan R., Senapong S., Puripattanavong J. (2014). In vitro antimicrobial and antibiofilm activity of *Artocarpus lakoocha* (moraceae) extract against some oral pathogens. *Tropical Journal of Pharmaceutical Research*.

[15] Hu W. S., Min Nam D., Kim J. S., Koo O. K. (2020). Synergistic anti-biofilm effects of *Brassicaceae* plant extracts in combination with proteinase K against *Escherichia coli* O157:H7. *Scientific Reports*.

[16] Dincer S., Masume Uslu F., Delik A. (2020). *Antibiotic Resistance in Biofilm*.

[17] Araya-Cloutier C., Vincken J. P., van Ederen R., den Besten H. M. W., Gruppen H. (2018). Rapid membrane permeabilization of *Listeria monocytogenes* and *Escherichia coli* induced by antibacterial prenylated phenolic compounds from legumes. *Food Chemistry*.

[18] El Atki Y., Aouam I., El Kamari F. (2019). Antibacterial efficacy of thymol, carvacrol, eugenol and menthol as alternative agents to control the growth of nosocomial infection-bacteria. *Journal of Pharmaceutical Sciences and Research*.

[19] Toma Z. (2010). Antimicrobial activity of piperine purified from *Piper nigrum*. *Journal of Basrah Researches*.

[20] Hikal D. M. (2018). Antibacterial activity of piperine and black pepper oil. *Biosciences Biotechnology Research Asia*.

[21] Zhang X., Li Q., Li H. (2021). High-level aminoglycoside resistance in human clinical *Klebsiella pneumoniae* complex isolates and characteristics of armA-carrying IncHI5 plasmids. *Frontiers in Microbiology*.

[22] Armbruster C. R., Parsek M. R. (2018). New insight into the early stages of biofilm formation. *Proceedings of the National Academy of Sciences*.

[23] Maldonado N. C., Silva De Ruiz C., Cecilia M., Nader-Macias M. E. F. (2007). A simple technique to detect *Klebsiella* biofilm-forming-strains. Inhibitory potential of *Lactobacillus* fermentum CRL 1058 whole cells and products. *Communicating Current Research and Educational Topics and Trends in Applied Microbiology*.

[24] Raei P., Pourlak T., Memar M. Y. (2017). Thymol and carvacrol strongly inhibit biofilm formation and growth of carbapenemase-producing gram negative bacilli. *Cellular and Molecular Biology*.

[25] Dwivedi D., Singh V. (2015). Effects of the natural compounds embelin and piperine on the biofilm-producing property of *Streptococcus mutans,*. *Journal of Traditional and Complementary Medicine*.

[26] Ramos L. D. P., Pereira T. C., Assis M. D. S. (2021). Antibiofilm activity of glycolic plant extracts on *Klebsiella pneumoniae* clinical isolates). *Research, Society and Development*.

[27] Deivamarudachalam Teepica P. D., Srinivasan P., Guna G., Manimekalai K., Jaganathan D. (2015). In vitro anti biofilm activity of *Piper longum* and *Piper nigrum* against clinical isolates of *Streptococcus pyogenes* isolated from pharyngitis patients. *International Research Journal of Pharmacy*.

[28] Roy R., Tiwari M., Donelli G., Tiwari V. (2018). Strategies for combating bacterial biofilms: a focus on anti-biofilm agents and their mechanisms of action. *Virulence*.

[29] Verderosa A. D., Totsika M., Fairfull-Smith K. E. (2019). Bacterial biofilm eradication agents: a current review. *Frontiers in Chemistry*.

[30] Kumrungsee N., Pluempanupat W., Koul O., Bullangpoti V. (2014). Toxicity of essential oil compounds against diamondback moth, *Plutella xylostella*, and their impact on detoxification enzyme activities. *Journal of Pest Science*.

[31] Stojanović-Radić Z., Pejčić M., Dimitrijević M. (2019). Piperine-a major principle of black pepper: a review of its bioactivity and studies. *Applied Sciences*.

